# Emergence of Selectivity to Looming Stimuli in a Spiking Network Model of the Optic Tectum

**DOI:** 10.3389/fncir.2016.00095

**Published:** 2016-11-24

**Authors:** Eric V. Jang, Carolina Ramirez-Vizcarrondo, Carlos D. Aizenman, Arseny S. Khakhalin

**Affiliations:** ^1^Department of Neuroscience, Brown UniversityProvidence, RI, USA; ^2^Biology Program, Bard CollegeAnnandale-on-Hudson, NY, USA

**Keywords:** looming detection, optic tectum, collision avoidance, recurrent networks, sensorimotor transformation, intrinsic excitability, homeostatic plasticity, visual development

## Abstract

The neural circuits in the optic tectum of Xenopus tadpoles are selectively responsive to looming visual stimuli that resemble objects approaching the animal at a collision trajectory. This selectivity is required for adaptive collision avoidance behavior in this species, but its underlying mechanisms are not known. In particular, it is still unclear how the balance between the recurrent spontaneous network activity and the newly arriving sensory flow is set in this structure, and to what degree this balance is important for collision detection. Also, despite the clear indication for the presence of strong recurrent excitation and spontaneous activity, the exact topology of recurrent feedback circuits in the tectum remains elusive. In this study we take advantage of recently published detailed cell-level data from tadpole tectum to build an informed computational model of it, and investigate whether dynamic activation in excitatory recurrent retinotopic networks may on its own underlie collision detection. We consider several possible recurrent connectivity configurations and compare their performance for collision detection under different levels of spontaneous neural activity. We show that even in the absence of inhibition, a retinotopic network of quickly inactivating spiking neurons is naturally selective for looming stimuli, but this selectivity is not robust to neuronal noise, and is sensitive to the balance between direct and recurrent inputs. We also describe how homeostatic modulation of intrinsic properties of individual tectal cells can change selectivity thresholds in this network, and qualitatively verify our predictions in a behavioral experiment in freely swimming tadpoles.

## Introduction

Spontaneous neural activity plays a key role in the developing nervous system. In the visual system of vertebrates, spontaneous activity generated both in the retina and in retinorecipient structures is critical for organizing early experience and facilitating the developmental refinement of neural circuitry (Pratt et al., 2016). However, spontaneous activity also places serious constraints on the normal processing of sensory stimuli by adding varying amounts of neural noise, and can therefore affect how the organism interacts with its environment. How then does a developing nervous system balance the need to sustain spontaneous activity with the ability to effectively process and react to external sensory stimuli? Here we examine the process of collision detection in Xenopus laevis tadpole tectum, in which activity generated through recurrent connectivity (Pratt et al., 2008; Liu et al., 2016) can interact with waves of evoked visual responses, potentially leading to differential reactions to different visual stimuli (Khakhalin et al., 2014).

Detection of visually expanding, or looming stimuli is ubiquitous across the animal world, and is critical for both navigation and predator avoidance (Sun and Frost, 1998; Preuss et al., 2006; Liu et al., 2011; Herberholz and Marquart, 2012; Vagnoni et al., 2012). Research suggests that different animals may rely on different types of computations to detect looming stimuli: it was proposed that in birds collision detection may be achieved through spatial integration of appropriately directed edge movements in different parts of the visual field (Frost and Sun, 2004). In other animals, such as adult Ranid frogs (Ishikane et al., 2005; Kuras et al., 2006; Kang and Li, 2010; Baranauskas et al., 2012), collision detection seems to rely on competitive temporal inactivation of inputs from OFF-detectors in a retinotopic system. Yet other species either combine spatial motion integration and competitive inactivation, as in the case of locusts (Gabbiani et al., 2002; Peron and Gabbiani, 2009; Fotowat et al., 2011), or have several distinct startle systems, some relying on motion processing, and some on visual OFF detectors, as it was described in fruit-flies (Card and Dickinson, 2008; Fotowat et al., 2009; de Vries and Clandinin, 2012; Schilling and Borst, 2015). For many popular experimental species however, including Zebrafish larvae and *Xenopus* tadpoles, the exact mechanisms of collision detection are not yet clear (Khakhalin et al., 2014; Temizer et al., 2015; Dunn et al., 2016). Similar to larval Zebrafish that perform three types of evasive maneuvers in response to different visual stimuli (Burgess and Granato, 2007; Bianco et al., 2011; Dunn et al., 2016), *Xenopus* tadpoles can execute either fast randomized escapes or slow course corrections (Khakhalin et al., 2014), suggesting that different competitive collision detection mechanisms may be at play.

As in Zebrafish (Dunn et al., 2016), detection of looming stimuli in tadpoles relies on the circuitry in the optic tectum (OT) (Dong et al., 2009; Khakhalin et al., 2014). It is known that principal neurons in the tectum receive strong recurrent excitation (Pratt et al., 2008; Liu et al., 2016) that supports spontaneous neuronal activity during development (Pratt and Aizenman, 2007; Imaizumi et al., 2013; James et al., 2015). Principal tectal neurons also demonstrate prominent and rapid inactivation of spiking (Aizenman et al., 2003; Ciarleglio et al., 2015), which allowed us to suggest that together these two phenomena may underlie, or at least contribute to collision detection (Khakhalin et al., 2014). We hypothesized that in the presence of strong recurrent connections, rapidly inactivating networks would naturally discriminate in favor of expanding stimuli, reminiscent of dendritic competition, and spike-frequency accommodation in looming-selective neurons in insects (Peron and Gabbiani, 2009). At the same time, strong recurrent connections may present a challenge for a behaving animal, as spontaneous recurrent activity can overpower both sensory inputs and computed pre-motor outputs. The developing brain is thus faced with the problem of finding a proper balance between recurrent and sensory inputs to each neuron, keeping spontaneous activity at the levels appropriate for circuitry development, and stimulus detection.

In this study we describe an informed spiking model of the *Xenopus* tectum based on the recent detailed cell-level description of this region (Ciarleglio et al., 2015), and test whether the activation of this network may support collision detection. As the exact topology of recurrent connections in the tectum is not known, we compare several hypothetical internal connectivity profiles, and make tentative predictions about which of these profiles are more likely to be utilized by real tadpoles. We also study how the relative strength of recurrent and sensory inputs affect generation of spontaneous activity and stimulus selectivity, and investigate the robustness of stimulus selectivity in recurrent networks to different levels of spontaneous neural noise.

## Results

### The computational model

**Figure 1:**
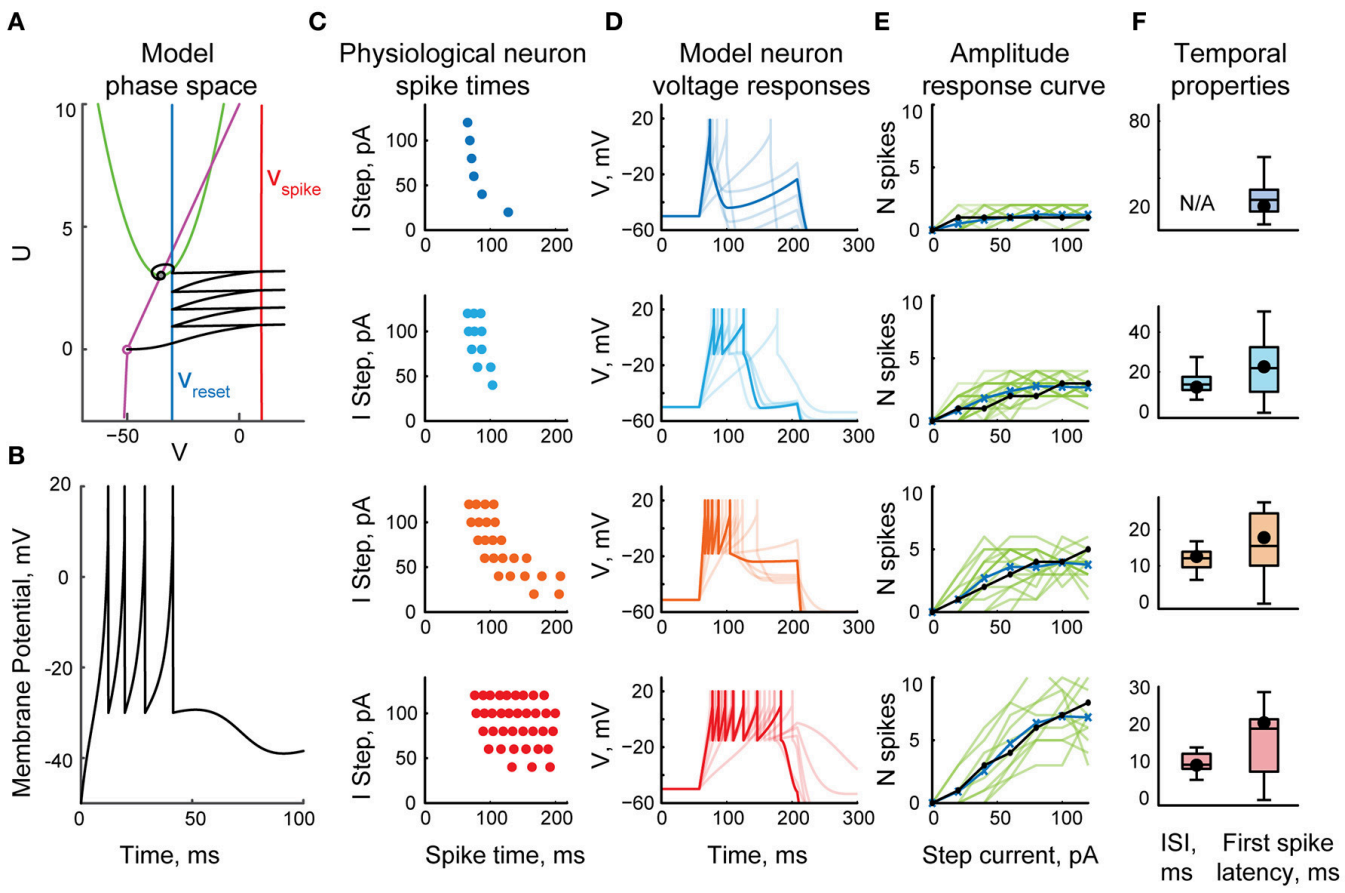
**The fast-inactivating spiking model of a tectal neuron. (A)** The phase space of a system of two differential equations representing a spiking neuron, showing a sample trajectory in this space (black), two nullclines (purple and green), and the values involved in potential reset during spiking (V_spike_ in red, and V_reset_ in blue). **(B)** A typical response of a model neuron to a step current injection. **(C)** Spike-time rasters of four representative physiological neurons from Ciarleglio et al. (2015) as they spike in response to current clamp steps of amplitudes from 20 to 120 pA. **(D)** Voltage traces of four model neurons in response to current steps of amplitudes from 20 to 120 pA. Responses to 80 pA current step are highlighted. **(E)** Input-output curves, showing the number of spikes generated by neurons in response to current step injections of different amplitudes, for four representative spiking groups separately. Response curves of individual biological neurons from Ciarleglio et al. (2015) are shown in green, averages for biological neurons in blue, model neuron responses in black. **(F)** Distributions of first spike latencies (left) and first-to-second inter-spike intervals (right) during responses of biological neurons to step current injections of 100 pA, with similar values for model neurons superimposed on them (black dots).

To keep the model computationally efficient, we represented each tectal neuron as a one-compartmental cell with spiking governed by a system of two ordinary differential equations: a quadratic differential equation with hard reset for voltage, and a linear differential equation for slow outward currents, similar to classic hybrid models with reset (Izhikevich, 2003, 2010). Compared to many other neural cells types however, principal neurons in the tadpole tectum typically produce very few spikes in response to both *in vitro* current injections (Ciarleglio et al., 2015) and *in vivo* visual stimulation (Khakhalin et al., 2014), yet show little frequency accommodation, presumably due to strong inactivation of Na^+^ voltage-gated channels. To approximate this spiking behavior, we adjusted the model by introducing several tuning parameters and a non-linear dependency between the input current in the cell and the change in cell potential (see Methods). These adjustments ensured that model neurons ceased spiking even in response to strong current injections (Figure 1A), and showed little frequency adaptation (Figure 1B).

To populate the network with appropriate cell types, we reanalyzed the data from Ciarleglio et al. (2015), and classified 104 biological cells recorded in stage 48–49 naïve tadpoles according to the maximal number of spikes they produced in response to step current injections. To simplify model tuning, we did not attempt to replicate the full gradient of neuronal excitability profiles, but classified biological cells from Ciarleglio et al. (2015) into four representative spiking phenotypes (Figure 1C): low-spiking cells that produced at most one spike; 3-spike cells (that produced 2 or 3 spikes); 5-spike cells (from 4 to 7 spikes), and highly spiking cells (8–11 spikes; 10 on average). For each cell group, we then collected three types of statistics: the number of spikes generated in response to whole-cell step current injections of different amplitudes (20–120 pA); the first spike latency in response to a 100 pA step current injection, and the inter-spike interval for 100 pA current injection. We then manually tuned four model neurons (Figure 1D), ensuring that they reproduce spike counts (Figure 1E), latencies, and inter-spike intervals (Figure 1F) observed in physiological experiments.

**Figure 2:**
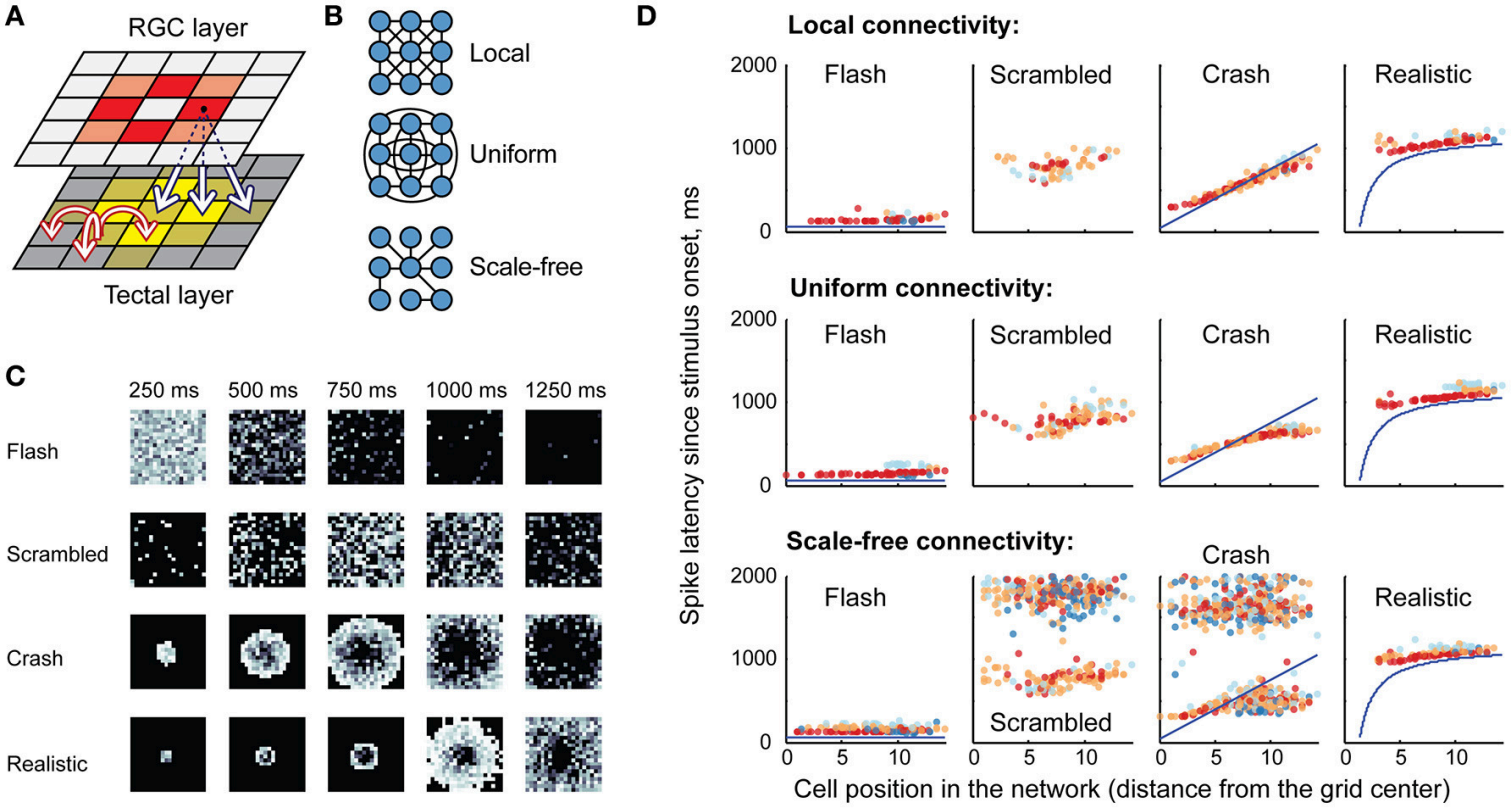
**Network model, retinal inputs, and network responses. (A)** The basic topology of the model network: a grid of retinal ganglion cells (RGCs) made a “blurred” retinotopic projection to the tectal layer (black arrows), with recurrent connections within the tectal layer (red arrows). Different colors schematically show relative activation of RGCs and tectal cells during visual stimulus processing. **(B)** Three possible options for the recurrent intra-tectal connectivity: local (neurons are connected only to their neighbors); uniform (connections between any two neurons are equally probable); and scale-free (small-world network with highly connected hub cells). **(C)** Snapshots of RGC layer spiking, representing four visual stimuli: instantaneous full-field **flash**; randomly rearranged (**scrambled**) looming stimulus; linearly expanding looming stimulus (**crash**); and **realistic** non-linearly expanding looming stimulus. Each square shows a “still frame” from a dynamic response, taken in 250 ms increments after the stimulus onset (0 ms); with more recent spikes within each 250 ms window shown in lighter shades of gray. **(D)** Sample rasters of spiking responses in the tectum, generated for different recurrent connectivity profiles (rows), and different visual stimuli (columns). The horizontal axis presents model cell positions as distance from the 20 × 20 grid center; vertical axis shows spike latency; blue lines for crash and realistic stimuli show the theoretical time at which each tectal cell is directly engaged by the visual stimulus through the corresponding retinal cell. Cells of different spiking phenotypes are shown in different colors, from most spiky (red) to least spiky (dark blue).

The model tectal network consisted of 400 cells arranged in a 20 × 20 grid; the cells were randomly assigned one of four cell types (1-, 3-, 5-, or 10-spike-generating cells) in the proportions observed in stage 49 tadpoles (20, 25, 40, and 15% respectively; Ciarleglio et al., 2015). This network received excitatory inputs from 400 retinal ganglion cells (RGCs) arranged in a similar grid. All RGCs were assumed to be OFF cells, and once activated, each RGC produced a train of four spikes with random inter-spike intervals; the distribution of these inter-spike intervals was adjusted to approximate both experimentally observed spiking of individual RGCs (Demas et al., 2012; Miraucourt et al., 2016), and bulk summary responses in the optic nerve during *in vivo* visual stimulation (Khakhalin et al., 2014). Projections from the RGC layer formed a “blurred” retinotopic map in the OT layer (Figure 2A), with a stride of 5 grid cells, and connection strength decreasing with distance.

As the topology of recurrent connections in the tadpole tectum is not known (Pratt et al., 2008; Liu et al., 2016), we considered three possible configurations (Figure 2B): **uniform**, with random connections across the entire network and uniformly distributed connection weights; **local**, with connections spanning 5 nearby cells in each direction, and with average strength decreasing with distance; and **scale-free**: a small-world network with a few strongly connected hub cells linking the entire network in a set of connected clusters (Barabasi and Albert, 1999). All connectivity profiles were normalized by synaptic strength, so that the average sum of synaptic inputs received by tectal cells was same in each network type, regardless of its topology. Both feedforward RGC-OT and recurrent OT-OT connections were modeled as conductance-based excitatory synapses with exponential decay, and dynamics approximating physiological data from Xu et al. (2011), Khakhalin et al. (2014), Ciarleglio et al. (2015). See Methods for more details on the model construction and parameter validation.

### Activation in response to visual stimuli

In computational experiments, the RGC layer simulated responses to virtual “visual stimuli” that were modeled after behaviorally relevant stimuli from Khakhalin et al. (2014). These included a full-field dark “**flash**”; a linearly expanding looming “**crash**”; a looming stimulus with its pixels randomly spatially rearranged on the 20 × 20 grid (“**scrambled**”), and a geometrically realistic looming stimulus with faster, non-linear hyperbolic dynamics (“**realistic**”). The spiking of RGC layer neurons over time is shown in Figure 2C.

In the tectal layer (Figure 2D) “flashes” evoked rapid, short responses, mostly mediated by high-spiking cells (red). “Scrambled” stimuli produced delayed and more prolonged responses with higher involvement of medium-spiky cells (orange and blue), but also lacking spatial organization, while “crashes” and “realistic” collisions created spatially organized waves of excitation that propagated through the tectum, from its center to the periphery. We observed that scale-free connectivity (bottom row of Figure 2D) easily gave rise to spontaneous epileptiform activity (clouds of points on the top of each raster plot, corresponding to ongoing spontaneous spiking), while activity in local and uniformly-connected networks tended to “die out” even in the absence of inhibition.

**Figure 3:**
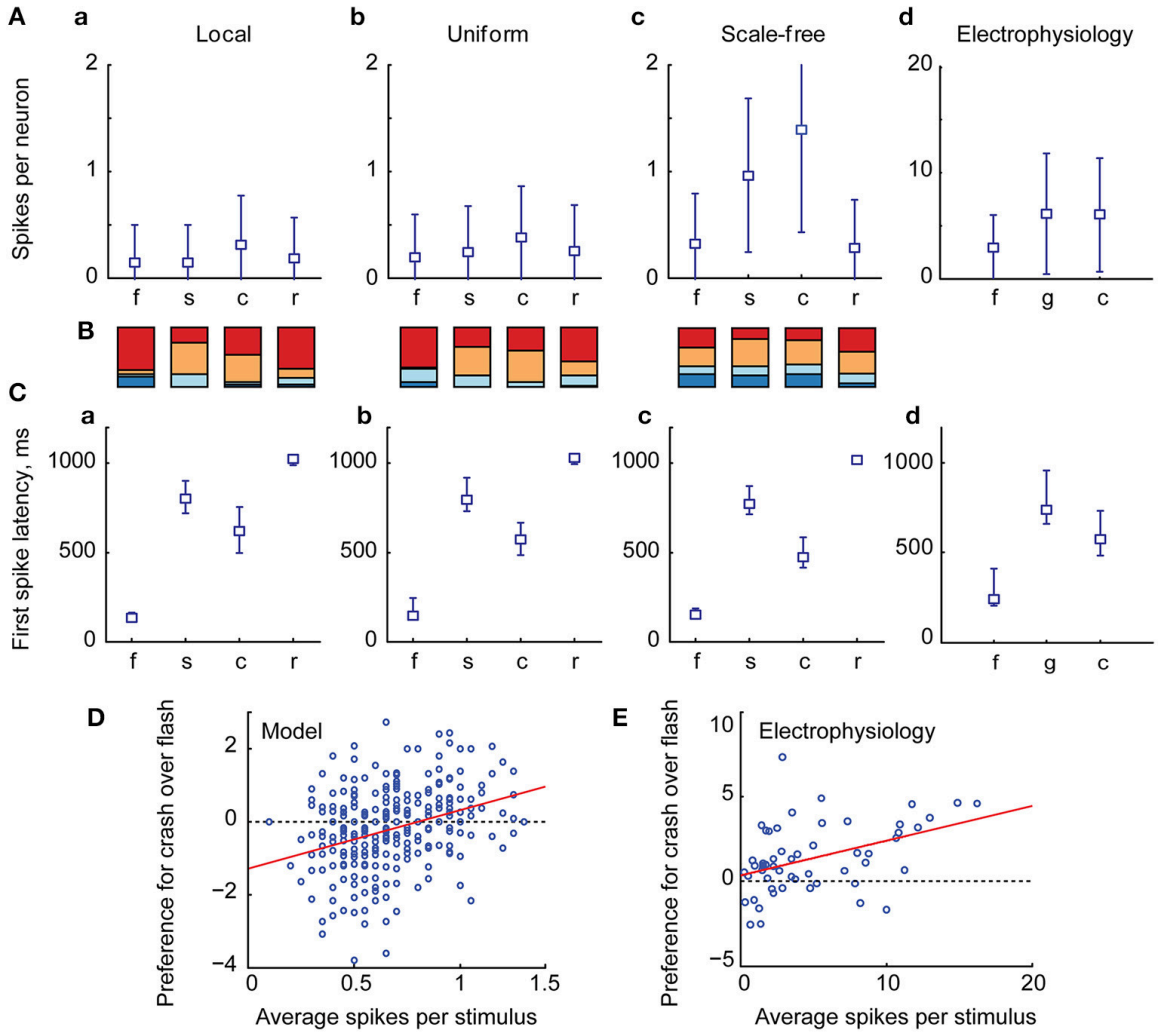
**The summary statistics of spiking output in the tectum. (A)** Averages (markers) and standard deviations (error bars) of the number of spikes per neuron generated by the tectum during responses to different visual stimuli, in models with different recurrent connectivity profiles **(A–C)**, and in physiological data [**D**, data from Khakhalin et al. (2014) Figure 4]. Here “f” stands for “flash,” “s” for “scrambled,” “c” for “crash,” “r” for realistic, and for physiological data “g” stands for “grid” (a stimulus that can be considered analogous to the “scrambled” stimulus from the model); responses were respectively modeled or recorded for 2s after stimulus onset. Both in computational and biological experiments looming stimuli evoked stronger responses than an instantaneous flash (paired *t*-test *p* = 5e−66, *n* = 400 for the model, *p* = 1e−5, *n* = 56 in physiological experiments; significant after Bonferroni correction), while spatially disarranged stimuli evoked intermediate responses **(B)**. The relative contribution of different neuronal spiking phenotypes to model responses, measured as the total number of spikes generated by all 10-spike (red), 5-spike (orange), 3-spike (light blue), and 1-spike (dark blue) neurons. Medium-spiking neurons were more involved in responses to slow than to fast stimuli **(C)**. The median and inter-quartile ranges of first spike latencies during tectal responses to different stimuli, in model networks with different connectivity profiles **(A–C)**, and in biological experiments **(D)**, data from Khakhalin et al. (2014), not previously presented). The model successfully predicted typical latencies observed in physiological experiments (all pairwise comparisons between responses to different stimuli *p* < 1e−6, paired *t*-test, significant after Bonferroni correction). **(D)** The average number of spikes generated by model neurons across all four visual responses correlated (*r* = 0.31) with their preference (Cohen d effect size) for looming stimuli (crashes) over flashes; regression line shown in red **(E)**. A similar analysis for the physiological data from Khakhalin et al. (2014) verified this prediction, as spikier neurons preferred looming stimuli to flashes (*r* = 0.42).

We found that the relative number of spikes generated in response to each type of stimulus (Figures 3A–C), as well as median latencies of neuronal responses (Figures 3A–C), matched the results of biological experiments (Figures 3A,C,D; data from Khakhalin et al. (2014). Linear looming “crashes” produced the highest total network activation, followed by spatially disorganized stimuli (“scrambled” in this study; “ramp” and “grid” in Khakhalin et al., 2014), and finally followed by synchronous “flashes.” The average number of spikes generated by neurons in the model was lower than that observed in physiological experiments (1.4 for “crash” in the model; 6.2 for “crash” in Khakhalin et al. (2014), for which we can offer several potential explanations (see Discussion).

The higher total spike-output in response to looming stimuli, compared to full field “flashes,” was primarily due to the stronger contribution of medium-high spiking neurons (orange in Figure 3B) that were mostly silent after “flashes,” but spiked in response to slower stimuli. In line with this observation, the relative preference for “crash” over “flash” in model data correlated with neuronal spikiness (Figure 3D). We verified this prediction by re-analyzing experimental data from Khakhalin et al. (2014), and found that in our *in vivo* experiments selectivity of individual neurons for “crash” over “flash” also correlated with their spikiness (Figure 3E, *r* = 0.4, *p* = 1e−3, *N* = 55; data from Khakhalin et al. (2014).

### Parametric analysis

We then investigated how the relative strength of recurrent connections and retinal inputs in the tectum affected network activation and selectivity for different visual stimuli. For each connectivity configuration we considered a family of models, with differently scaled weights of direct and recurrent inputs. Each network model was defined by two scaling coefficients: SR for RGC inputs, and ST for recurrent intratectal connections. With SR = 1 and ST = 0 there were no functional recurrent currents, and all drive to tectal cells came from RGCs, reaching on average 180 pA at peak (a rather high number, compared to *in vivo* average peak value of 70 pA in Xu et al. (2011), and 30 pA in Khakhalin et al. (2014). Conversely, with SR = 0 there were no inputs from the retina, while with SR = ST the average strengths of direct and recurrent connections were equal.

**Figure 4:**
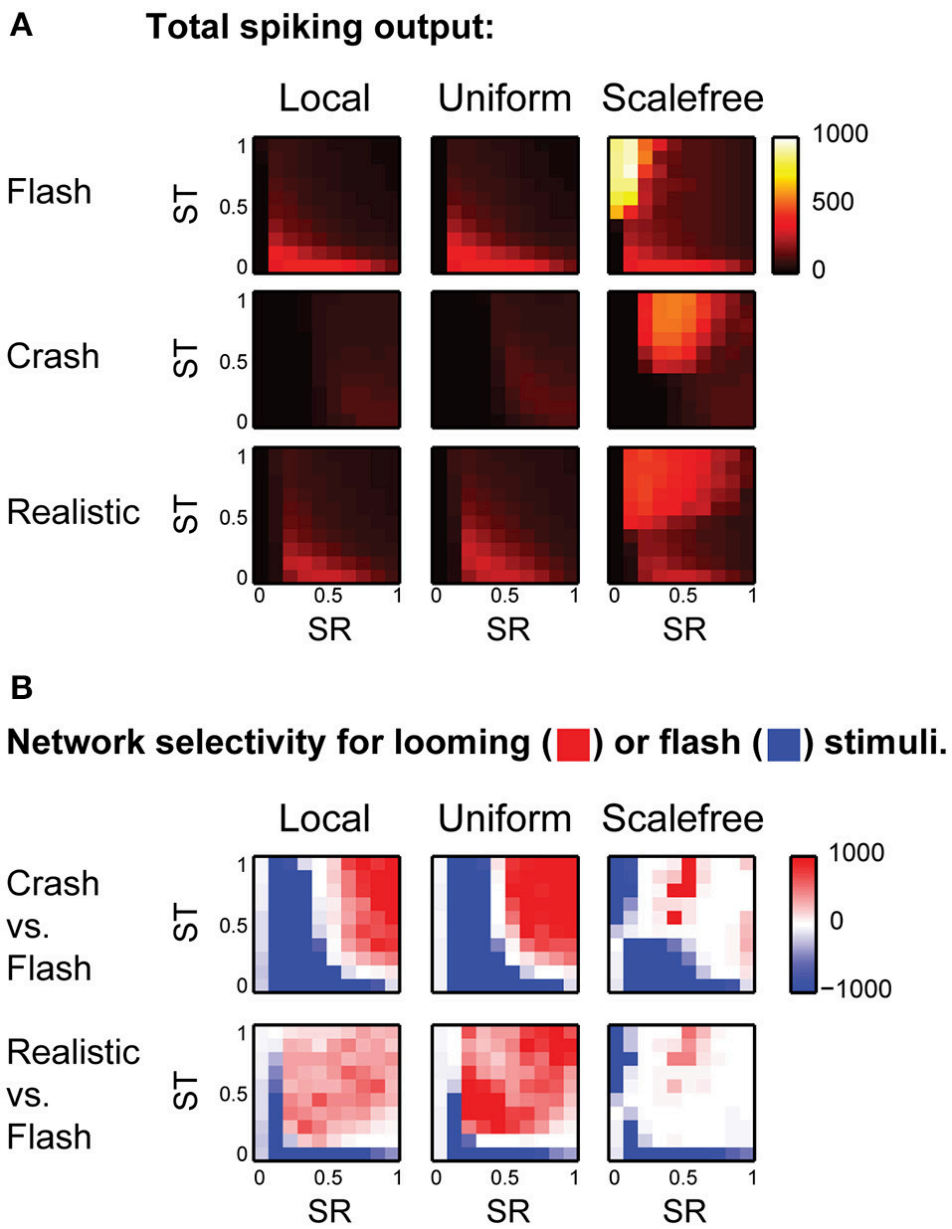
**The effect of balance between recurrent and direct inputs to the tectum on stimulus selectivity (A)**. The total number of spikes (shown as pixels of different color, from black to white) generated in model networks with different strength of direct (horizontal axes) and recurrent (vertical axes) synaptic inputs, for different stimulus types (rows), and recurrent network topologies (columns) **(B)**. The comparison of looming stimuli responses to full-field flash responses. Here color encodes the reliability (signed *F*-value) of getting a stronger total network response to either looming (red), or flash (blue) stimulus, for different strengths of direct (SR) and recurrent (ST) synaptic inputs. Networks with strong direct and recurrent synaptic inputs are selective for looming stimuli (red in the top right corner), while weakly connected networks are selective for full-field flashes (blue crescents in the left lower corner). This pattern is present, but less pronounced for fast looming stimuli, and in scale-free networks.

We ran the model 25 times for each combination of SR and ST, and calculated total amount of spikes generated in response to each stimulus in each experiment. The average values of total spike output are shown at (Figure 4A), encoded by color, with lighter colors representing stronger spike outputs. We then used signed *F*-values to quantify statistical reliability of stimulus preference across multiple experimental runs, and compared responses to different visual stimuli for each point in the (SR, ST) parametric space (Figure 4B). This calculation divided the (SR, ST) space into regions statistically selective for “flashes” (shown in blue in Figure 4B); selective for looming stimuli (red), and non-selective regions (white). We found that for both “local” and “uniform” recurrent connectivity profiles most of the parametric space was selective to looming stimuli (red in Figure 4B), while models with underpowered RGC-OT or OT-OT synaptic connections were selective for full field flashes (blue crescents in bottom left corners in Figure 4B). The parametric space for “scale-free” connectivity looked somewhat similar, but less selective, as networks were easily overpowered by epileptiform activity (of a kind shown earlier in Figure 2D, bottom row). The effects of spontaneous epileptiform activity can also be seen in respective spike-output heat maps (Figure 4A, third column), as yellow and orange areas of high total spiking at the top of each heat map (corresponding to regions of strong recurrent connectivity, ST > 0.5). Overall, our results suggest that in the absence of neuronal noise, the balance between recurrent and direct inputs in tectal networks does not have to be tight, as the networks are naturally selective to looming stimuli, provided that both direct and recurrent inputs are strong enough.

### Effects of spontaneous neuronal noise

**Figure 5:**
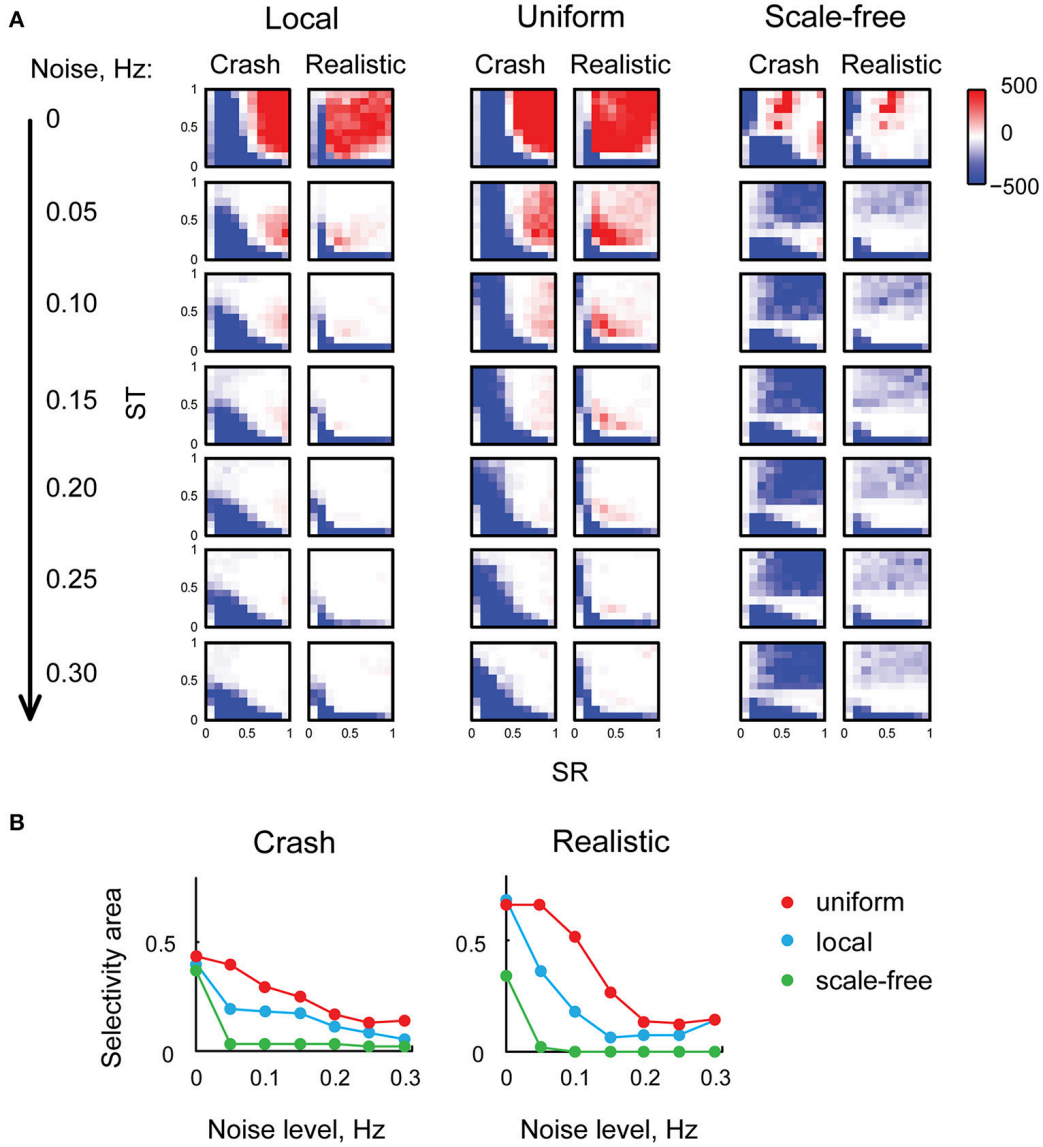
**Neuronal noise reduces selectivity to looming stimuli, and makes the balance of direct and recurrent inputs more important. (A)** Selectivity charts in the (SR, ST) space for different connectivity profiles and two types of looming stimuli (columns), shown for different levels of spontaneous neural noise (rows). As the levels of noise increase (top to down across the panel), the areas of selectivity for looming stimuli become smaller. **(B)** The share of the parametric space selective to looming stimuli (with arbitrary threshold of *F* = 10), as a function of neural noise level, for two types of looming stimuli. The share of (SR, ST) combinations allowing looming stimuli detection goes down as noise levels increase, yet uniformly connected network (red) is less sensitive to background noise than either local (blue) or scale-free (green) networks.

We then investigated the sensitivity of looming stimuli detection in our model to the level of background neural noise. We made every tectal neuron in the model spontaneously fire action potentials at frequencies from 0 to 0.3 Hz, and analyzed these “noisy” networks in the same way as we analyzed the original network. The results of these experiments are presented in (Figure 5A): as the level of neuronal noise increased (from top rows down in Figure 5A), red areas, representing tuning parameter combinations that kept the network selective for looming stimuli, shrunk, and then disappeared altogether. From the shape of red looming-selective regions in the second and third rows of (Figure 5A) we can see that for moderate levels of neuronal noise (0.05–0.10 Hz) collision detection happened only when the strength of recurrent connections offered a trade-off between the absence of temporal integration for low values of ST, and susceptibility to epileptiform spontaneous activity for large values of ST. Moreover, as the levels of noise increased, the areas of selectivity for slow “crashes” and fast “realistic” collisions became increasingly non-overlapping, suggesting that for high levels of neuronal noise the balance of recurrent and direct connections may be important not just for enabling collision detection, but also for tuning it to specific temporal dynamics of the stimulus.

To quantify the range of “valid” (SR, ST) combinations that kept the network tuned for looming detection under conditions of high neuronal noise, for each heat map from (Figure 5A) we measured the relative size of the looming-selective region in the (SR, ST) parametric space, using an arbitrary threshold of 10 for the *F*-value. We found (Figure 5B) that selectivity areas reduced in size with increasing levels of neuronal noise, and that the uniformly connected network (red) was most robust, followed by locally-connected network (blue), while scale-free network was very sensitive to noise due to its propensity to spontaneous activity (green). These results suggest that in realistic conditions of non-zero neuronal noise, the balance of recurrent and direct inputs in the tectum may require a tight homeostatic control, and that the level of inflexibility in tuning increases with the amount of spontaneous activity in the system.

### Effects of sensory experience

**Figure 6:**
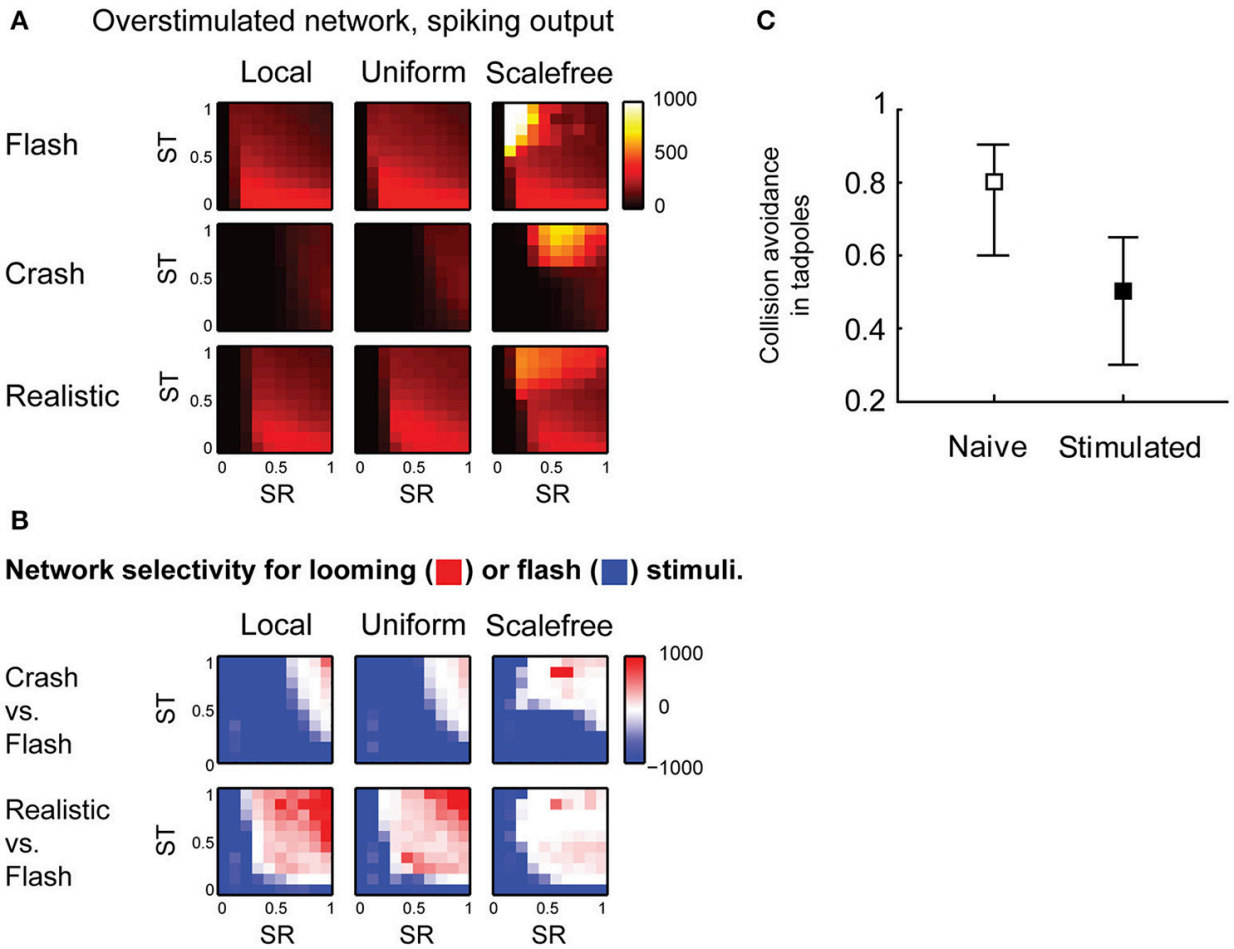
**Effects of sensory experience on looming stimulus detection. (A)** Compared to naïve networks shown in Figure 4, overstimulated networks spike more in response to fast stimuli (“flash” and “realistic” looming), but not in response to slow “looming” stimuli. Color shows the total number of spikes generated by the network in response to a stimulus **(B)**. Unlike naïve networks, overstimulated networks are not selective for slow looming stimuli (large blue areas in the first row), but retain selectivity for fast “realistic” looming stimuli (red areas in the second row) **(C)**. In behavioral experiments, after prolonged strong visual stimulation tadpoles don't perform avoidance maneuvers in response to slow-moving objects (Mann-Whitney *P* = 0.003).

Finally, we looked into the effects of sensory experience on spontaneous activity and collision detection in the tectum. We updated the model to mimic the changes in the tectum of *Xenopus* tadpoles in response to strong, prolonged visual stimulation (Aizenman et al., 2003; Ciarleglio et al., 2015), and analyzed this “overstimulated” tectal network in the same way as we analyzed the “naïve” network. We changed the distribution of spike phenotypes to 5, 30, 20, and 45% for 1, 3, 5, and 10-spike-generating cells respectively (Ciarleglio et al., 2015); decreased all synaptic currents by 25% (Aizenman et al., 2002); and introduced 30% inward rectification for synaptic currents (Aizenman et al., 2002). The parametric space analysis showed that in overstimulated networks the selectivity for slow “crashes” was lost, except for areas of extreme synaptic strength (Figure 6A), while selectivity for fast collisions remained largely unaffected (Figure 6B).

From these results, we predicted that after prolonged stimulation with light flashes tadpoles would retain avoidance of fast collisions, but would no longer respond to slow moving objects. We performed a series of behavioral experiments in freely swimming stage 49 Xenopus tadpoles, and found that after prolonged visual stimulation the avoidance of large (11 mm in diameter) slow (1.4 cm/s) black circles was indeed significantly impaired (from 0.73 ± 0.22 to 0.51 ± 0.23, Mann-Whitney *P* = 0.003; Figure 6C), while it was previously shown, using a slightly different, but conceptually similar experimental protocol, that the avoidance of smaller (8 mm) black circles moving at faster speeds (3 cm/s) is not affected by prolonged sensory stimulation (Dong et al., 2009).

## Discussion

In this study we show that a simple experimentally inspired retinotopic network of inactivating spiking neurons can naturally function as a detector of looming stimuli, provided that recurrent excitation in the network is strong enough, and the network is not overpowered by spontaneous neural noise. Notably, this selectivity for looming stimuli occurs even in the absence of inhibition, and without contribution from specialized motion or expansion detectors. Importantly, our model replicates some of the key physiological and behavioral features of looming stimulus detection in the tectum of *Xenopus* tadpoles, potentially including effects of strong visual stimulation on collision avoidance.

Our results indicate a possibility of functional convergent evolution between looming detection in vertebrates and insects, as in our model a retinotopic layer of rapidly inactivating neurons seemed to discriminate looming stimuli in a way that is fundamentally similar to how spike frequency adaptation and competitive inhibition make it happen in the dendritic tree of looming-selective DCMD neurons in locusts (Gabbiani et al., 2002; Peron and Gabbiani, 2009). In both cases, inactivation-like processes (recurrent excitation and sodium channel inactivation in *Xenopus*; spike frequency adaptation and spatially localized shunting inhibition in insects) introduce a competition mechanism that discriminates against stimuli that are either too synchronous and localized or develop too slowly. To evoke the highest total spike-output, in both insects and tadpoles, the sensory input should develop as a wave of increasing strength, running slow enough to allow temporal summation (Gabbiani et al., 2004; Khakhalin et al., 2014), yet fast enough to get ahead of competitive inactivation in the retinotopic system (Peron and Gabbiani, 2009; Jones and Gabbiani, 2010). These local inactivation mechanisms therefore may link temporal and spatial properties of the sensory stimulus, providing a simple and efficient mechanism for stimulus recognition.

The discrepancy between the average number of spikes observed in this model study (about one or two) and during physiological *in vivo* loose cell-attached recordings (5–12 spikes; Khakhalin et al., 2014), which happened despite a careful and meticulous calibration of cell intrinsic properties we undertook in this study, can be explained by several possible effects. One potential difference between the studies of neuronal spiking in whole cell (Ciarleglio et al., 2015) and loose cell-attached modes (Khakhalin et al., 2014) is that spike-triggering currents arrive at different compartments within the neuron: in the soma in case of whole-cell studies, but at the dendritic tree during synaptic stimulation. Because of that, our model, which was based on the data from Ciarleglio et al. (2015), might have underestimated the effects of active dendritic integration that may in fact take place in the optic tectum (Bollmann and Engert, 2009; Felch et al., 2016). Another complication of whole-cell studies is that during the recording, messenger molecules may be washed out from the cytoplasm of the neuron (Zhang et al., 1998; Khakhalin and Aizenman, 2012), potentially triggering changes in intrinsic properties and loss of spikiness (Staley et al., 1992). Although in Ciarleglio et al. (2015) intrinsic properties were typically recorded first, immediately following the entry to the cell, this effect might have contributed to lower estimations of cell spikiness in this study. Further, during data acquisition for (Khakhalin et al., 2014), cells that were silent to both “crash” and “flash” after one or two presentations were not included in the analysis, introducing a selection bias, which was not present in this study.

Our study suggests that looming stimulus detection can be supported by very different recurrent connectivity profiles, including spatially disorganized random uniform networks. It may mean that at least for the purposes of collision detection, the refinement of recurrent connections in the tectum may be not as critical as the refinement of input retinotectal projections (Dong et al., 2009), although it may still be critical for computing appropriate directional motor responses during avoidance of slow-moving objects, as described in Khakhalin et al. (2014). The best robustness to spontaneously generated neural noise was observed in a spatially unorganized uniformly connected network, which intuitively lies “in between” a local recurrent network and a scalefree network in terms of how strongly on average it connects neural cells from different parts of the model tectum. (A formal justification for this claim can be achieved through a calculation of the average distance between two randomly selected nodes in the network of *N* nodes, which is typically the largest for a locally connected network (it grows with *N* as a power of *N*), intermediate in a random network (grows slower, as ln *N*), and is the smallest (grows as ln ln *N*) in the scale-free network we used (Barabási, 2014, ch. 4, p. 21). In effect, for connectivity profiles, as well as for synaptic scaling parameter ST that defined the strength of recurrent connections, it was the “middle solution” that offered most robust collision detection under conditions of moderate neural noise, while both weakly connected and hyperconnected networks failed. This finding suggests that developing tectal networks in *Xenopus* tadpoles may employ a yet undescribed homeostatic mechanism to balance relative abundance and strength of direct and recurrent synaptic connections in principal tectal cells, keeping it in the range that supports collision avoidance computations. Our modeling data also suggests that collision detection may be further improved by recurrent feedback inhibition (Khakhalin et al., 2014; Liu et al., 2016), especially for networks that are tightly connected and susceptible to epileptiform activity, or when the level of spontaneous neuronal noise in the system is high. We hypothesize that delayed feedback inhibition may act as a safeguard, temporally limiting recurrent activity in the network, and thus allowing for strong integration between direct and recurrent inputs within the window before the inhibition onset, without the risk of succumbing to epileptiform activity.

Overall, we demonstrated that recurrent networks with inactivation can indeed underlie collision detection, and that appropriate calibration and tuning of recurrent and direct inputs to the tectum becomes more important as the levels of noise generated by spontaneous activity in the network increase.

## Methods

### Spiking cell model

Our spiking cell model is governed by two ordinary differential equations: a quadratic differential equation with hard reset for voltage (V), and a linear differential equation for slow outwards currents (U):

(1) $$\{\begin{array}{l} \frac{dV}{dt}=\frac{1}{C}\;\cdot \;\left(k_{1}\left(V-V_{r}\right)\left(V-V_{th}\right)-U+IM\right)\\ \frac{dU}{dt}=a(k_{2}\left(V-V_{r}\right)-U) \end{array}$$

Here *V* represents cell membrane potential (the value of *V* is dimensionless, but can be interpreted as membrane potential in mV); *U* (also dimensionless) approximates both activation of slow K+ channels and inactivation of Na+ channels; *V*_*r*_ stands for resting membrane potential (typically −50); *V*_*th*_ represents voltage-gated Na+ channels threshold potential (*a*-value in the −5 to −20 range, depending on the cell type); *C* is a tuning parameter similar to cell membrane capacitance (large values of *C* make cells spike slower); *I* is the external current injected in the cell (in pA), and *M* represents the current adjustment for leak and space clamp effects.

The parameter *a* controls the speed of inactivation (U), and flips between two values, depending on whether the voltage is increasing or decreasing, to better represent the dynamics of recovery of physiological neurons from Na channels inactivation, as observed in Ciarleglio et al. (2015) during responses to cosine current injections:

(2) $$a=\{\begin{array}{cc} a_{1},\; & \text{if}\;\frac{dV}{dt}>0\\ a_{2},\; & \text{otherwise} \end{array}$$

Of parameters *k*1 and *k*2, the latter was made dynamically dependent on the value of external current injected in the cell (I):

(3) $$\begin{array}{l} k_{1}=\frac{-4L}{{\left(V_{th}-V_{r}\right)}^{2}}\\ k_{2}=\;\left\{ \begin{array}{l} \begin{array}{cc} b & \text{ if }2\cdot \frac{L+I}{V_{th}-V_{r}}>b\\ 0.2 & \text{ if }2\cdot \frac{L+I}{V_{th}-V_{r}}<0.2 \end{array}\\ \begin{array}{cc} 2\cdot \frac{L+I}{V_{th}-V_{r}} & \text{otherwise} \end{array} \end{array}\right. \end{array}$$

Here *L* and *b* are tuning parameters. With these adjustments, compared to the original Izhikevich model (Izhikevich, 2003), the parabolic and linear nullclines of the phase portrait (Figure 1A) always intersect: as the parabolic nullcline moves up during positive current injections, the linear nullcline changes its slope, always passing through the lower point of the parabola. As a result, the phase space retains a stable attractor for the phase trajectory, ensuring that even for high currents the neuron never switches to regular spiking, but generates a limited near-constant number of spikes (Figure 1B). Moreover, unlike in bursting and thalamo-cortical varieties of the Izhikevich model, where fast inactivation is achieved by an increase in the value of U, our neurons showed very little spike-frequency adaptation, with almost constant inter-spike intervals within a burst, as it is the case in real physiological neurons (Ciarleglio et al., 2015).

The equations were solved using an Euler method with time step *dt* of 0.1 ms, except for the hard spiking reset that was implemented algorithmically:

(4) $$\text{if}\;V>V_{spike},\;\text{then}:\;\;\{\begin{array}{c} \text{​​​}V\leftarrow V_{reset}\\ U\leftarrow U+d \end{array}$$

where tuning parameter *d* contributes to inactivation speed. Here and below, all modeling and analysis of results were performed in Matlab (MathWorks Ltd., Natick, MA).

### Cell model tuning and calibration

We manually tuned four model spiking cells to represent electrophysiological subtypes of tectal cells observed in Ciarleglio et al. (2015). We also selected a representative physiological cell for each group to serve as a general visual guide (cell ids: 28,003, 9004, 1104, and 9003 from Ciarleglio et al. (2015) respectively). For each of four cell types, our goal was to make the model match as well as possible the mean number of spikes for different injected currents, and the median latency and inter-spike intervals, or at least be within one standard deviation from it. The results of this manual tuning process are shown in Figures 1E,F.

To make sure that model cells respond adequately to dynamic inputs, we compared spiking of model and biological cells in response to cosine current injections of different frequencies (data not shown, but see (Ciarleglio et al., 2015) for details of the protocol). As each model cell spiked in a deterministic fashion, we had to introduce noise in each of the tuning parameters before visually comparing average behavior of model cells to that of biological cells. This rough comparison prompted us to introduce different speeds for spiking inactivation and recovery (parameters *a*_1_ and *a*_2_ in the model above), as depolarization-associated inactivation of spiking in biological cells was typically much slower than recovery during in-between hyperpolarization periods.

The final set of model parameters for the four representative cell types were chosen as follows:

**Table:** 

**Parameter**	**1-spike cell**	**3-spike cell**	**5-spike cell**	**10-spike cell**
1/C	0.1036	0.0451	0.0444	0.0513
V_r_	−50	−50	−51.1765	−50
V_th_	−20	−14.1176	−6.353	−14.8235
V_spike_	9.5294	10	10	10
V_reset_	−12	−12	−18.0588	−15.0588
M	0.34	0.5918	1.3406	0.5682
a_1_	0.022	0.02	0.0068	0.0106
a_2_	0.33	1.2	0.374	0.848
L	−1.4118	−2.3824	−8.0294	−3.2647
d	50	20.6	31.4	6.1579

### Network population

The model tectal network consisted of 400 cells arranged in a 20 × 20 grid. A fixed share of these cells was assigned each of the four subtypes, according to the actual distribution of spikiness in biological stage 49 tadpoles (Ciarleglio et al., 2015): 20%, 25%, 40% and 15% respectively for naïve animals (default state of the network, Figures 2–5), and 5%, 30%, 20% and 45% for the “overstimulated” network (Figure 6). The cell types were randomly permuted for each model run.

### Synaptic transmission

Synaptic connections were modeled as an excitatory conductance-based transmission with exponential decay of conductance over time after each pre-synaptic spike:

(5) $$\frac{dG_{i}}{dt}=-\frac{G_{i}}{\tau }+q\;\cdot \;\sum_{j}w_{ij}S_{j}$$

(6) $$I_{i}=G_{i}\cdot \left(E-V\right)$$

where *G*_*i*_ represents synaptic conductance of cell *i* at this moment of time; *S*—the vector of pre-synaptic cell spike-trains, with each spike treated as a delta-function, *w*—the matrix of synaptic input weights (synaptic strengths); *q*—scaling sensitivity of this neuron type to synaptic inputs (set to 2.5, 2, 1.5, and 1.5 for neurons of 4 spiking types respectively); τ—synaptic conductance decay (25 ms); I—synaptic current; E—excitatory reversal potential (0 mV), and V—cell membrane potential at this moment of time. We found that with these values of synaptic parameters, the model produced subthreshold postsynaptic potentials that were very similar in shape to average postsynaptic potentials observed in biological experiments in response to synchronous activation of visual inputs (Ciarleglio et al., 2015). We also found that in response to suprathreshold synchronous synaptic stimulation of biologically reasonable strength, our model cells on average produced the same number of spikes (from 1 to 10) that they produced in response to current injections. As dynamic properties of recurrent intra-tectal synapses are not yet described in the literature, we modeled them in the same way as synapses from the retina. Our model did not include effects of short-term pre-synaptic plasticity, such as paired-pulse facilitation or paired-pulse depression.

For the model of overstimulated tectal network (Figure 6) synaptic transmission was further adjusted: all synaptic conductances (*q*) were reduced by 25%, and inward rectification was introduced (Aizenman et al., 2002), reducing synaptic currents by further 30% if the postsynaptic cell has a positive membrane potential:

(7) $$\{\begin{array}{ll} I_{i}=0.7\;\cdot \;G_{i}\left(E-V\right), & \text{if }V>0\\ I_{i}=G_{i}\left(E-V\right), & \text{otherwise} \end{array}$$

### Projections from RGCs to OT

The model retina consisted of 400 “retinal ganglion cells” (RGCs), arranged in a 20 × 20 square matrix, and producing trains of spikes in response to “visual stimulation” (see below). Each RGC cell was connected to a square spanning 5 cells in each direction from its “precise projection” in the tectal retinotopic network. It led to a total projection size of about one half tectal network width, roughly matching projection size in real *Xenopus* tectum (Shen et al., 2011). The strength of synaptic inputs within this projection square was inversely proportional to the Euclidian distance between each tectal cell location (*i, j*) and the “precise projection” point (*ip, jp*):

(8) $$w_{RT}=\frac{w_{max}}{1+\sqrt{{\left(i-ip\right)}^{2}+{\left(j-jp\right)}^{2}}}$$

### OT recurrent connectivity

In the model tectum, we tested three different connectivity profiles: uniform connectivity, in which each tectal cell was equally probable to get connected to every other tectal cell; local, in which only neighboring tectal cells were connected, and scalefree, which gave the model properties of a small world network. All three connection profiles were calibrated to deliver similar total drive to the tectal cells (see below).

For the **uniformly connected** OT network, we first created a random adjacency (connectivity) matrix, with every tectal neuron connected to every other tectal neuron, and synaptic weights *w*_*ij*_ uniformly distributed between 0 and 1. We then removed self-connections, calculated the sum of all inputs received by each tectal cell (total synaptic drive), and divided all input weights for this cell to its total synaptic drive, thus scaling the total sum of inputs received by each cell to 1:

(9) $$\text{for}\;\text{each}\;\text{i}:\;\;\;\sum_{j}w_{ij}\;=\;1$$

For the **locally connected** OT network, we first connected all neurons randomly and uniformly, as described above, and removed all self-connections. We then scaled all weights between tectal neurons based on the Euclidian distance between them along the rectangular grid, making the average weight linearly increase from zero for cells separated by 5 or more grid steps, to strong connections between immediately neighboring cells:

(10) $$w=\xi \;\cdot \;\{\begin{array}{ll} 1-\frac{D}{5},\; & \text{if }D\leq 5\\ 0,\; & \text{otherwise} \end{array}$$

were *D* is the distance between cells, $D=\sqrt{{\left(x_{1}-x_{2}\right)}^{2}+{\left(y_{1}-y_{2}\right)}^{2}}$, and ξ is a random variable uniformly distributed on [0, 1]. The width of 5 cells in each direction was chosen to match the width of retinotectal connections seen in Shen et al. (2011), and seems to roughly replicate the direct measurements of tectal activation observed after local release of glutamate in the tectum (Carlos Aizenman, unpublished data), which is the best guess we can make in the absence of published observations. In the same way as it was done for the uniform network, we then calculated the total sum of inputs received by each cell, and scaled inputs by this number, ensuring that each cell in the network received the same total synaptic drive, regardless of the size and position of its recurrent connectivity “watershed.”

For the **scale-free** tectal network, we followed the version of Barabasi algorithm (Barabasi and Albert, 1999) as implemented in the SFNG Matlab script (George, 2006). After the scale-free connection graph was constructed, the weights of all established connections were randomized with a uniform distribution between 0 and 1, and scaled (normalized) for each cell in the same way as for uniform and local connectivity profiles.

### Balancing retinal and recurrent inputs

As relative strength of direct and recurrent inputs to tectal cells in real *Xenopus* tecta are not known, we created a family of model networks with different average strengths of retinotectal and recurrent inputs, and performed a hyperparameter analysis for this family of models. We multiplied all normalized synaptic weights by two scaling factors: SR for retinal inputs, and ST for tecto-tectal recurrent inputs. For SR = 1 the total synaptic current in each model cell during full-field synchronous visual stimulation reached 180 pA, which was 2–3 times higher than highest amplitudes of total synaptic currents recorded *in vivo* in *Xenopus* tadpole tectum (Xu et al., 2011; Khakhalin et al., 2014), and was similar to strongest synaptic currents ever recorded in tectal cells in response to optic chiasm stimulation (160–250 pA in selected cells in Ciarleglio et al. (2015). As in our model, for SR = 1 this extreme current was experienced by all cells in the tectum, we could be sure that for SR = 1 the retinal inputs were too strong (stronger than in a biological tectum), and so the “realistic” SR value would lie somewhere between 0 and 1.

As isolated recurrent tectal currents are not well studied in the biological tectum, we linked recurrent scaling coefficient ST to the value of SR, in such a way that for ST = SR the total recurrent drive experienced by tectal cells during massive spiking in the tectum would be on average the same as for the retinotectal sensory drive. A practical interpretation of ST values is therefore similar to that for SR: for ST = 0 all recurrent connections were eliminated, while for ST = 1 recurrent connections were obviously exaggerated, suggesting that the unknown “realistic” value of ST would lie somewhere in the [0, 1] region. For Figures 2, 3 we used values of SR = ST = 0.5, which produced visual responses similar to that in Khakhalin et al. (2014), and spontaneous recurrent events similar in strength to spontaneous barrages in James et al. (2015). For hyperparameter search in Figures 4–6, the values of SR and ST were sampled between 0 and 1 in steps of 0.1.

### Visual stimuli

The model retina was presented with four different black-and white (binary) virtual visual stimuli. In case of instantaneous full-field **flash**, the entire visual field went black at moment *t* = 0. For **crash**, or linear looming stimulus, a black circle grew from the center of the visual field and onto the edge, with the radius of the circle linearly increasing with time over a course of 1 s, as in Khakhalin et al. (2014). For **scrambled**, first a crash stimulus was calculated, and then 400 pixels of which it consisted were randomly rearranged (we used a different random permutation in each computational experiment, but the permutation was fixed during the experiment itself). Finally, a **realistic** stimulus was also looming, but with the relative radius of the visual stimulus increasing hyperbolically, to reproduce a perspective projection during a frontal collision with a flat object:

(11) $$R=\sqrt{2n}\;\cdot \;\frac{1-v}{1-vt}$$

where *n* is the number of RGC neurons (400); *v* is a dimensionless approach speed (0.1), and time *t* changed from 0 to 1 s.

### RGC spiking

All retinal cells (RGCs) were modeled as “off” cells, with simple one-pixel receptive fields, together representing the 20 × 20 virtual black-and-white visual stimuli. The change of a virtual pixel from white to black triggered a “response” in the corresponding RGC. Each response consisted of 4 spikes, reflecting the average number of spikes recorded in loose cell-attached recordings from individual RGCs in Xenopus tadpoles (Demas et al., 2012; Miraucourt et al., 2016). The latency of the first spike was distributed normally with the mean of 50 ms and standard deviation of 17 ms, while the inter-spike intervals followed a gamma-distribution with mean of 50 ms and standard deviation of 20 ms. With these parameter values, the superposition of spike-trains generated by the model retina in response to full-field flashes well reproduced the average response in the optic nerve in response to full field stimulation (Khakhalin et al., 2014).

### Spontaneous activity

When studying the effects of noise on stimulus selectivity (Figure 5) we also introduced background spontaneous spiking in the tectal network. In these computational experiments each cell was equally likely to generate a “spontaneous spike” at each time tick, with frequencies ranging from 0 to 0.3 Hz. These “spontaneous spikes” were not modeled fully, and did not affect the instantaneous membrane potential V of the cell itself, but triggered postsynaptic potentials in all cells that received innervation from the spiking cell, according to the weight of this connections, in the same way as it would have happened for a “normal,” evoked spike. Note that this approach to modeling of spontaneous neuronal noise may somewhat exaggerate the network effects of it, as it does not take into account the inactivation of sodium channels after each spontaneous spike. We also made all neuronal types generate the same amount of spontaneous spikes, even though in a biological network high-spiking neurons may generate more spontaneous activity than low-spiking neurons.

### Analysis

For the analysis of looming stimulus selectivity regions in the (SR, ST) parametric space (Figures 4–6), we ran the model 25 times for each recurrent connectivity profile, stimulus type, and the combination of SR and ST parameters. In each run all other parameters of the network were randomized (cell types assignments, synaptic connectivity weights, RGC spiking patterns, and background spiking, where applicable). For each model run we calculated the total number of spikes generated by the network during 2s of visual stimulus processing (Figures 4A, 6A), and used signed *F*-values (the share of variance in total spiking responses *S*, explained by the stimulus type as a factor, taken with the sign of average response difference between two stimuli) as a measure of statistical reliability of looming stimulus selectivity:

(12) $$\begin{array}{l} F=\text{sign}\left(\bar{S_{1}}-\bar{S_{2}}\right)\cdot \frac{explained\;variance}{unexplained\;variance}=\\ \;=\text{sign}\left(\bar{S_{1}}-\bar{S_{2}}\right)\cdot \frac{var\left(\left[\begin{array}{cc} \bar{S_{1}} & \bar{S_{2}} \end{array}\right]\right)}{var\left(\left[\begin{array}{ccc} S_{1}-\bar{S_{1}} & , & S_{2}-\bar{S_{2}} \end{array}\right]\right)}\cdot \frac{N-1}{N-2} \end{array}$$

where *S*_1_ and *S*_2_ are random variables representing total network responses to two different stimulus types, each value obtained in a different model run; square brackets stand for vector concatenation (as in Matlab notation), and *N* is the total sample size for values compared:

(13) $$N=length\left(S_{1}\right)+length\left(S_{2}\right)=25\;+\;25=50$$

For the analysis of noise influence (Figure 5) we used an arbitrary threshold of *F* = 10 to classify noisy networks with different scaling parameters combinations as either selective for looming stimuli or not, similar to how it is shown as differently colored regions in Figure 4B.

When Cohen d effect size is reported, it was calculated as *d* = Δ*m*/*s*, where Δ*m* is the difference of means, and *s* is a pooled standard deviation across both groups.

### Behavior

All animal experiments were performed in accordance with Brown University Institutional Animal Care and Use Committee standards, and were approved by the committee. For behavioral experiments, *Xenopus* tadpoles were raised as described previously (Ciarleglio et al., 2015) until they reached developmental stage 48–49 (Nieuwkoop and Faber, 1994). At this point they were either put to experiment directly from the incubator (“naïve” group), or were first stimulated by lines of green LEDs flashing in sequence at 1 Hz for 4 h (Aizenman et al., 2003; Dong et al., 2009; Ciarleglio et al., 2015). One by one, tadpoles were then placed in a Petri dish, and each of them was presented with a black circle 11 mm in diameter projected on the floor of the chamber. Every 30 s the circle was sent toward the tadpole at a speed of 1.4 cm/s, to elicit a collision avoidance response, as described in Khakhalin et al. (2014). Tadpole behavior was recorded with a video camera, tracked in Noldus EthoVision XT (Noldus Information Technology, Leesburg, VA, USA), and analyzed offline. Each tadpole was presented with 8–10 stimuli (average of 9.8), and avoidance responses were counted. Trajectory analysis showed that neither average distance (1.5 ± 0.7 cm), nor average speed of successful avoidance responses (4.0 ± 3.8 cm/s) were different between naïve and overstimulated tadpoles (Mann-Whitney *p* = 0.8 and 0.2 respectively), suggesting that we observed a true change in collision detection and avoidance responsiveness in the sensory and sensorimotor regions of the brain, and not a difference in avoidance maneuver implementation.

## Author contributions

EJ and AK designed the model and ran the computational experiments. CR designed, ran and analyzed the behavioral experiments. CA and AK oversaw the project, contributed to the overall experimental design and interpretation. All authors contributed to the manuscript.

### Conflict of interest statement

The authors declare that the research was conducted in the absence of any commercial or financial relationships that could be construed as a potential conflict of interest.
